# Intestinal epithelial cell metabolism at the interface of microbial dysbiosis and tissue injury

**DOI:** 10.1038/s41385-022-00514-x

**Published:** 2022-05-09

**Authors:** Eva Rath, Dirk Haller

**Affiliations:** 1 Technical University of Munich, Chair of Nutrition and Immunology, Freising-Weihenstephan, Germany; 2 Technical University of Munich, ZIEL Institute for Food & Health, Freising-Weihenstephan, Germany

## Abstract

The intestinal epithelium represents the most regenerative tissue in the human body, located in proximity to the dense and functionally diverse microbial milieu of the microbiome. Episodes of tissue injury and incomplete healing of the intestinal epithelium are a prerequisite for immune reactivation and account for recurrent, chronically progressing phenotypes of inflammatory bowel diseases (IBD). Mitochondrial dysfunction and associated changes in intestinal epithelial functions are emerging concepts in the pathogenesis of IBD, suggesting impaired metabolic flexibility of epithelial cells affects the regenerative capacity of the intestinal tissue. Next to rendering the intestinal mucosa susceptible to inflammatory triggers, metabolic reprogramming of the epithelium is implicated in shaping adverse microbial environments. In this review, we introduce the concept of “metabolic injury” as a cell autonomous mechanism of tissue wounding in response to mitochondrial perturbation. Furthermore, we highlight epithelial metabolism as intersection of microbiome, immune cells and epithelial regeneration.

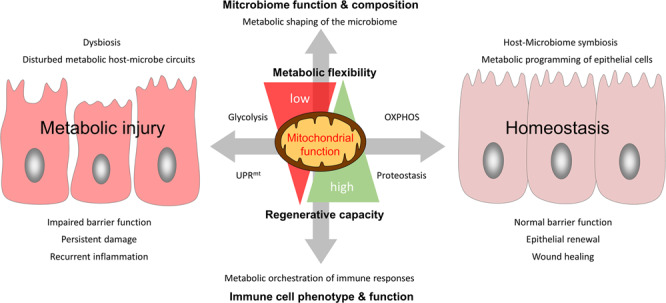

## Introduction - from energy deficiency to chronic inflammation

Inflammatory bowel diseases (IBD) are a paradigm for the complex interplay of gene-environment interactions in the development and progression of immune-mediated pathologies, and together with metabolic disorders, their incidence and prevalence are increasing, following a pattern of industrialization^1,2^. Crohn’s disease and ulcerative colitis are the two main clinical phenotypes^3^ and dysbiotic changes of the intestinal microbiome emerged as a common link between host genetic susceptibility (>240 variant alleles)^4,5^ and environmental cues^6,7^. Crohn’s disease is characterized by discontinuous and transmural inflammation predominantly affecting the ileo-colonic part of the intestine, while ulcerative colitis is restricted to the colonic mucosa. These anatomically and functionally distinct areas of the intestinal tract create spatially adapted microbial habitats, likely contributing to the heterogeneity of disease phenotypes in IBD. The intestinal epithelium represents the frontline of the complex pathogenesis, lying at the interface of luminal inflammatory triggers such as the microbiome and host immune cells, and a breach of this well-structured barrier is suggested as cornerstone of chronic inflammation. Consequently, episodes of tissue injury and incomplete healing of the intestinal epithelium are a prerequisite for immune reactivation and account for recurrent, chronically progressing phenotypes of IBD. Injury-associated stem cells are imperative in orchestrating tissue regeneration, and dynamic adaptations of mitochondrial metabolism in the intestinal stem cell (ISC) niche are essential to ensure tissue homeostasis. Thus, these results support the concept in which a reduced metabolic flexibility of IECs affects the regenerative capacity of the epithelium and renders the intestinal mucosa towards increased susceptibility to inflammatory triggers^8^. Mitochondria are increasingly recognized as site of microbial signal-integration, and microbiome-derived metabolic signals emerge as an important player in determining the ability for mucosal healing^9–11^. Intermittent flares of mucosal inflammation are associated with rapid individual changes in the microbiome of Crohn’s disease patients, and relapsing as well as remitting Crohn’s disease phenotypes are transmissive via fecal transfer in germ-free mouse models^12^, supporting the hypothesis that functional alterations in the microbiome not only contribute to the progression but also to the remission of disease.

W.E. Roediger proposed in 1980 that ulcerative colitis is an energy deficiency disease of the intestinal epithelium defined by diminished butyrate oxidation leading to alterations in mucus synthesis, and crypt cell maturation^13^. In the inflamed tissue microenvironment of IBD patients, infiltrating immune cells, together with the energy requirements of resident epithelial and stroma cells limit the available oxygen^14^, and together with a reduced blood supply, these changes contribute to hypoxic conditions in chronic inflammation^15^. Interestingly, mitochondrial dysfunction and associated changes in intestinal epithelial functions are suggested as early event in the pathogenesis of IBD, preceding inflammatory tissue aberrations^8,16–21^. In particular, impaired mitochondrial function in intestinal epithelial cells (IEC) is associated with reduced stemness and Paneth cell dysfunction^18,20,22^. However, even under inflammation-associated hypoxia, the remaining oxygen in the intestinal tissue is sufficient to support mitochondrial oxidative phosphorylation (OXPHOS)^15^, and these changes cannot explain the complex and early (before onset of histological inflammation) alterations in epithelial metabolism observed in intestinal inflammation^13,18,20^. It seems an intriguing hypothesis that alterations in epithelial cell oxidative metabolism based on disturbed mitochondrial functions is not only a consequence, but rather a causal factor in the development of chronic intestinal inflammation^23–25^. First hints towards this idea came from genome-wide association studies mapping IBD genetic risk loci to mitochondrial function-associated genes^26–28^, implying mitochondrial disturbances as underlying mechanism in IBD pathogenesis.

**Fig. 1:**
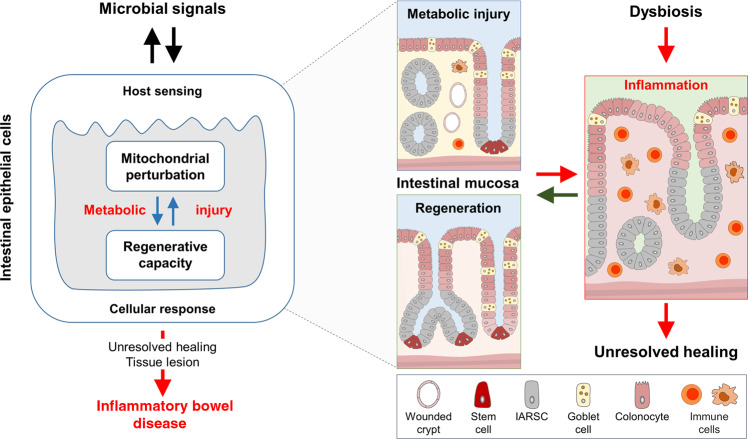
The novel concept of metabolic injury as underlying mechanism of recurrent inflammation in patients with inflammatory bowel diseases (IBD). Mitochondrial perturbation of the colonic epithelium e.g., caused by a combination of genetic predisposition and environmental triggers results in tissue injury (referred to as metabolic injury). Unresolved metabolic injury and incomplete tissue regeneration take place at the intersection of mitochondrial stress signaling (UPRmt) and changes of epithelial metabolism driving microbial dysbiosis and chronic inflammation in patients with IBD. UPRmt mitochondrial unfolded protein responses, IARSC injury-associated regenerative stem cells.

In this review, we introduce the concept of “metabolic injury” as a cell autonomous mechanism of organ and tissue wounding in response to mitochondrial perturbation (Fig. 1). By accounting for unresolved tissue injury, metabolic injury might play a key role in the pathogenesis of inflammatory and tumorigenic disorders of the digestive tract. Furthermore, we highlight epithelial metabolism as intersection of microbiome, immune cells and epithelial regeneration.

## Intestinal epithelial architecture and metabolism

The intestinal epithelium is a multicellular interface located in close proximity to a complex and dense microbial milieu. Simultaneously intestinal epithelial cells absorb nutrients, and form a physical and immune-mediated barrier against adverse components of the luminal environment. The intestinal epithelium represents the most regenerative tissue in the human body highlighted by the fact that self-renewal of this single cell-layered interface (30–40 m^2^) is completed every 3–5 days, most likely as a protective mechanism against injuries and infections. Under homeostatic conditions of self-renewal but also in response to injury, crypt base columnar cells (CBCs) expressing the leucine-rich repeat-containing G protein-coupled receptor 5 (Lgr5) feed the transit amplifying zone of the epithelium, and progenitor cells finally differentiate into mature secretory (e.g., Paneth cells, goblet cells, enteroendocrine cells) and absorptive cells (columnar shaped enterocytes)^29^. Olfactomedin 4 (*Olfm4*), is an alternative marker for highly proliferative intestinal stem cells, co-expressed with, but not restricted to Lgr5^+^ stem cells. Numbers of Olfm4 positive cells expands during mucosal healing processes^30^, and are implicated in colorectal cancer^31^. The Olfm4 gene shows an abnormal methylation and expression patterns in ulcerative colitis^32^, and encodes a putative secreted glycoprotein. Yet, Olfm4 is also involved in the regulation of mitochondrial respiration and cellular ATP level^33^. Paneth cells contribute to stem cell homeostasis and anti-microbial defence exclusively in the small intestine, while goblet cells are spread throughout the entire epithelial lining of the digestive tract, and specifically in the colon, these secretory cells produce a thick and well-structured mucus layer responsible for the sequestration of bacteria from the host^34^. Loss of Lgr5^+^ stem cells in response to severe (often transmural) injury requires additional regenerative capacities in the intestine, involving quiescent slow-cycling cells located at the +4 position in the crypt, de-differentiated epithelial cells and reserve stem cells^35^. A breach in barrier integrity is associated with inflammatory pathologies^36^, and requires immediate action to restore barrier function and to induce mucosal healing^37^.

Metabolism, long perceived as mere supplier of ATP, is increasingly appreciated to reflect and determine cellular phenotypes. The central metabolic pathways, including mitochondrial OXPHOS, glycolysis, tricarboxylic acid (TCA) cycle, pentose phosphate pathway, fatty acid oxidation, fatty acid synthesis and amino acid metabolism, are tightly interrelated and essentially contribute to the availability of biosynthetic precursors and energy. Beyond constituting “building blocks”, central components of intermediary metabolism are co-factors or co-substrates of chromatin-modifying enzymes, and metabolic enzymes are directly involved in the control of gene expression^38^. Thus, metabolism and gene expression employ a regulatory interface, and consequently, IEC differentiation requires distinct metabolic identities, characterized by highly regulated changes in mitochondrial activity. Crypt cells including the stem cell niche and transit amplifying cells mainly rely on glycolysis for ATP generation, whereas differentiation and maturation of intestinal epithelial cells is accompanied by increased dependence on mitochondrial OXOHOS to meet their energetic needs. This metabolic gradient along the crypt-villus axis and is reflected by the mitochondrial content of the cells, which also determines cellular levels of reactive oxygen species (ROS). The balance of ROS scavenger systems and ROS produced by the mitochondrial respiratory chain control the activation of mitochondria-dependent apoptotic cascades in senescent epithelial cells, hence mitochondrial functions steer and coordinate epithelial renewal and cell shedding^39^. Not only mitochondrial metabolism, but also mitochondrial proteostasis and the associated mitochondrial unfolded protein response (UPR^mt^) contribute to these processes and are involved in intestinal pathologies by controlling self-renewal and the proliferative capacity of the epithelium^22,40^. This is not only critical to maintain epithelial homeostasis, but also crucial during inflammation and for dedifferentiation processes associated with wound healing. Both requires a tight regulation of cell proliferation and cell death programs and hence, mitochondrial functions. Furthermore, mitochondrial metabolism is a driving force in the generation of wound-associated epithelial cells^41^ a cell type required for efficient barrier restoration upon injury. Thus, shifts in epithelial metabolism under inflammatory conditions might partly represent changes in IEC subtype composition and differentiation state and therefore, be secondary to inflammation. Vice versa, genetic risk factors affecting cellular metabolism might render particularly IECs sensitive to environmental triggers of inflammation, and thereby impair the regenerative capacity of the epithelium.

In the light of these findings, the functional plasticity of the intestinal epithelium and its regenerative response to injury are modulated or even controlled by mitochondrial metabolism, supporting the hypothesis that mitochondrial exhaustion contributes to the functional perturbation of the epithelium in IBD (referred to as metabolic injury).

## Mitochondrial signaling in the epithelium

Mitochondria play a profound role as platforms sensing the cellular environment and eliciting appropriate responses to cope with physiological disturbances. For example, mitochondria contribute to inflammatory processes by production of ROS^42^, and mitochondrial DNA can act as damage-associated molecular pattern (DAMP), promoting inflammation through toll-like receptor 9-dependent mechanisms^43^. Yet, also mitochondrial metabolic activity itself and the associated signaling pathways constitute critical checkpoints for ensuring epithelial integrity in the intestine^39^. Unfolded protein responses (UPR) are cornerstones in the homeostatic regulation of organelle function^44^, first described for the endoplasmic reticulum and implicated in the pathogenesis of IBD^45,46^. Mitochondrial UPR is essential in maintaining cellular metabolism and function^47^, but under conditions of intestinal inflammation, the consequences of sustained mitochondrial stress in the epithelium are completely unclear. Highlighting the importance of mitochondrial proteostasis for intestinal homeostasis, overexpression of prohibitin 1, a mitochondrial chaperone for proteins belonging to the electron transport chain in intestinal epithelial cells^48^, or therapeutic delivery of prohibitin 1 via nanoparticles^49^ protected mice from chemically induced colitis. Vice versa, conditional deletion of the mitochondrial chaperone heat shock protein 60 (*Hsp60*) in intestine^22^ and in liver^50^ gave rise to the global concept that cell autonomous perturbation of mitochondrial function requires paracrine mediators to cause tissue injury. Using mouse models in which loss of Hsp60 causes UPR^mt^ activation and subsequent mitochondrial dysfunction specifically in intestinal stem cells (ISC) or IECs, we illustrated the crucial role of mitochondrial metabolism for intestinal stemness, Paneth cell functionality and the proliferative capacity of IECs^18,22,51^. Deficiency in mitochondrial OXPHOS upon deletion of Hsp60 causes loss of Lgr5+ ISCs and emergence of non-granular, dysfunctional Paneth cells, accompanied by tissue aberrations reminiscent of mucosal wound healing^18,22^. Importantly, mitochondrial-dysfunction associated aberrances of the intestinal stem cell niche were not only observed in mouse models of intestinal inflammation, but also in a cohort of Crohn’s disease patients^18^. In this prospective patient cohort, presence of mitochondrial dysfunction-associated markers in the intestinal stem cell niche in non-inflamed tissue margins could stratify the risk of disease recurrence^18^, suggesting that metabolic perturbations in the crypt epithelium precede inflammatory tissue lesions.

A number of IBD-related genetic risk loci map to mitochondrial function-associated genes, supporting the idea that mitochondrial perturbations and limited metabolic flexibility sensitize the intestinal epithelium to additional insults^26–28^. For example, *SLC22A5* encodes the carnitine transporter OCTN2 involved in mitochondrial fatty acid oxidation^52^. *IRGM* affects mitochondrial fission^53^, a process crucial for the dynamic adaptation of mitochondrial function to physiological cellular changes^54^. Another IBD risk allele involved in the regulation of *PPIF* (encoding Cyclophilin D)^4^ affects cell death mechanisms by controlling mitochondrial membrane potential and the mitochondrial permeability transition pore (MPTP)^55^. Mitophagy removes dysfunctional mitochondria and several genetic risk variants are associated with this cellular process. Among these are *PARK7*^56^, *SMURF1*^57^, *LRRK2*^58^, but also prominent IBD-related genes including *ATG16L1*^59,60^ and *NOD2*^61,62^. Overall, a recent analysis found that 5% of IBD susceptibility genes identified have direct roles in regulating mitochondrial homeostasis^56^. Of note, also mitochondrial-encoded DNA polymorphisms are associated with ulcerative colitis^63,64^, and experiments using conplastic mouse strains (possessing identical nuclear DNA but distinct mitochondrial DNA) suggest that increased mitochondrial OXPHOS and ATP levels protect from experimental colitis^65^. In summary, changed expression of mitochondrial genes and proteins, mitochondriopathy, perturbed mitochondrial dynamics, mitochondrial dysfunction, and activation of mitochondrial stress signaling were observed in IBD patients^39,66,67^, and added new facets to the old “energy deficiency” hypothesis (reviewed in^9,68^). Consequently, mitochondrial metabolism and signaling including the UPR^mt^ have been implicated in integrating nutrient- and microbiota-derived signals in the intestinal stem cell niche^8^, critically affecting the regenerative capacity of the epithelium under homeostatic and disease conditions.

## Metabolic circuits between the microbiome and the intestinal epithelium

The human digestive tract harbors a complex array of microorganisms, including bacteria, archaea, viruses and fungi^69^. Bacteria colonize the compartmentalized gastrointestinal tract in a spatially structured manner following a gradient from the proximal to the distal part of the intestine, reaching highest density and functional diversity in the colon^70^. In comparison to the small intestine, the motility of the colon is substantially slowed down leading to a prolonged retention of luminal content (20–50 h) and the accumulation of biologically active metabolites^71^. In this context, the term microbiome describes the “theatre of activity” including the complex physio-chemical characteristics of the microbial communities within the niche shaped by the host^72^. Disruption of this microbiome-host symbiosis contributes to the initiation and progression of immune and metabolic diseases, such as IBD^73^, graft-versus-host diseases (GvHD)^74^, and type 2 diabetes^75^, underlining the proposition that microbe-host interactions are critically important for human health^76^.

**Fig. 2:**
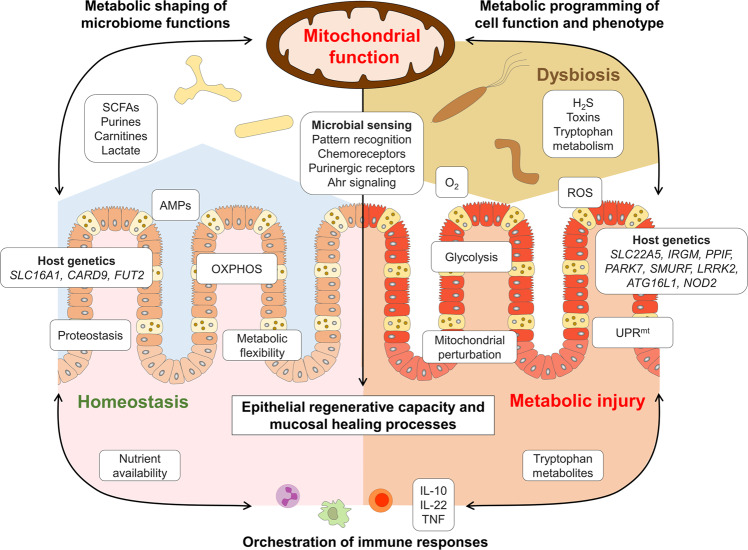
Intestinal epithelial mitochondrial function as intersection of microbiome, immune cells and epithelial regeneration. The intestinal epithelium senses the microbial environment via pattern recognition receptors and receptors sensing metabolites. Left: Under homeostatic conditions, intestinal epithelial cell (IEC) mitochondrial function contributes to the selection of a beneficial microbiome by maintaining low luminal oxygen concentration through oxidative phosphorylation (OXPHOS) and supporting production of antimicrobial peptides (AMPs). The microbiome provides metabolic support of epithelial cells by fermentation products such as short chain fatty acids (SCFAs), lactate, purines, and carnitines, thereby promoting cellular energetics and metabolic flexibility of IECs. As the ability to adapt mitochondrial functionality to the cellular demand determines the epithelial regenerative capacity, perturbations of mitochondrial metabolism result in metabolic injury of the epithelium (right). Shifting cellular metabolism away from OXPHOS to glycolysis (leading to elevated O2 levels) and impaired AMP production might result in dysbiosis, in turn aggravating the pro-inflammatory environment by reducing beneficial metabolites/ increasing disadvantageous microbial functions. IECs suffering from mitochondrial perturbation are exposed to high levels of reactive oxygen species (ROS) and activate mitochondrial stress signaling pathways such as mitochondrial unfolded protein response (UPRmt). Host genetics impact the selection of microbiota (left) and mitochondrial functions (right). IEC metabolism in conjunction with microbiota-derived metabolites likely controls mucosal immune cell recruitment and differentiation, thus orchestrating healing responses. Vice versa, immune cell-derived factors such as cytokines steer epithelial responses by targeting mitochondrial functions and metabolism. IL interleukin, TNF tumor necrosis factor.

Several pathogens and their toxins specifically target and disrupt mitochondrial function^77^, and vice versa, mitochondrial stress in IECs increases bacterial translocation^78^. Yet, the mucosal interface not only responds to infections, but also to non-pathogenic bacteria^79–81^. This finding seeded the idea that intestinal tissue homeostasis requires active engagement of multifaceted microbial (such as pattern recognition receptors)^82^ and chemical sensors^83^ (including olfactory receptors and purinergic receptors (ATP receptors)^84–87^) and implicated the microbiome not only contributing to IBD progression but also to confer protective mechanisms. A main paradigm in IBD pathogenesis is that mucosal tolerance towards the “normal” microbiota is lost, and interestingly, mitochondrial dysfunction impairs the ability of the intestinal epithelium to be tolerant to commensal bacteria^62^. On the other hand, the microbiome provides metabolic support for the epithelium, including butyrate^13^, lactate^88^, and purines^89^ (Fig. 2). Thus, the microbiome can modulate ISC niche function and host metabolism through direct contact or release of products/ metabolites^90,91^. Studies in germ-free mice demonstrated a profound effect of the microbiome on amino acid, glutathione and overall energy metabolism^92,93^, as well as on IEC-maturation and differentiation^94,95^. Of note, expression of the lactate and butyrate transporter solute carrier family 16 member 1 (*SLC16A1*/ MCT1) is reduced in ulcerative colitis, along with genes encoding enzymes of the mitochondrial β-oxidation pathway^96^, indicating that reduced availability of substrates might arise from bacterial alterations as well as cellular disturbances. Consequently, loss of metabolic circuits is associated with the development of chronic inflammation^18,21,40,66,67,97^ and implicate a mitochondrial (genetic) susceptibility-plus-microbiome function interaction in IBD pathogenesis. Intestinal epithelial mitochondria might be key to initiation of inflammatory processes, as they are integral to epithelial homeostasis and at the same time, are exposed to beneficial and detrimental luminal factors. In line, several microbiome-derived mediators have been identified that enhance epithelial regeneration by sustaining cellular energetics and are depleted under inflammatory conditions.

## Microbiome-derived metabolic signals and mucosal healing

Fecal stream diversion in patients with active Crohn’s disease and fecal microbial transplantation^98–100^ provided first clinical evidence to support the hypothesis that the intestinal microbial milieu contributes to disease recurrence^101,102^. Microbiome alterations are evident in the initiation and progression of disease activity, and considering the high energy demand of the inflamed mucosa, the (disturbed) interplay of epithelial metabolic functions and the microbial milieu might be of particular relevance in IBD^67^.

Disturbed metabolic circuits between microbiome and host, including butyrate^13^, carnitine^103^, purine^89^ and tryptophan metabolism^104^, are involved in the regulation of epithelial regenerative capacity and the development chronic inflammation, supporting the idea that mitochondrial perturbations contribute to IBD pathogenesis^18,21,40,66,67,97^. IECs are highly adapted to their anatomical location. Hence, small intestinal enterocytes predominantly utilize glucose and glutamine for energy generation, while microbiota-derived short-chain fatty acids (SCFA) represent the major energy source for colonocytes^39^.

There have been numerous reports indicating reductions in SCFA-producing bacteria in IBD^105^. However, substantial inter-individual variations are reported for SCFA levels^106^, and it is unlikely that the reductions in SCFAs observed under inflammatory conditions result in a primary energy deficiency of colonocytes. Under healthy conditions colonic concentrations of butyrate range from 10 to 20 mM^107^ (70–100 mM for the SCFAs acetate, propionate and butyrate combined^108^), and portal vein plasma concentrations range from 14–20 µM^109,110^, despite metabolization rates of 70–90% given for butyrate in colonocytes^108,111^. Plasma concentrations of acetate and propionate are even higher than those of butyrate^110^, indicating a spillover of SCFAs into the blood stream and suggesting SCFA availability to exceed the physiological energy demand of colonocytes.

Furthermore, SCFAs regulate PGC1α, a major transcription factor of mitochondrial biogenesis, and other genes involved in energy metabolism while promoting IEC growth^112–114^. Consistently, colonocytes from germ-free mice show a metabolic shift from OXPHOS to glycolysis^93^ and concomitantly, diminished cell cycle progression^115^. These effects were associated with perturbed pyruvate dehydrogenase (PDH) function, and could be rescued by supplementation of butyrate^115^. Of note, the metabolic effects were attributed to butyrate serving as energy substrate and not butyrate acting as inhibitor of HDAC activity^93^, which has been reported to inhibit proliferation of colonic ISCs via Foxo3^116^. Propionate and acetate are additional substrates for the TCA cycle and ameliorate metabolic and intestinal diseases by activating intestinal gluconeogenesis (like microbiome-derived succinate^117^)^118^ and by enhancing innate immune responses via free fatty acid receptor 2 (FFAR2)-signaling^119^.

Similarly, lactate serves as energy substrate fueling OXPHOS, thereby enhancing intestinal stemness^120^. Microbiome-derived lactate furthermore accelerates colonocyte turnover^121^ and by activating Gpr81 either on Paneth cells or on stromal cells, augments Wnt factor-production, resulting in Lgr5^+^ ISCs expansion and protection from acute intestinal damage^88^. Recently, also microbiome-derived purines have been identified as checkpoint metabolites, critically modulating cellular energetics, proliferation, and epithelial barrier function^122^. Purines like microbiome-derived hypoxanthine allow efficient biosynthesis of nucleotides and IECs can improve their energy balance by preferentially salvaging exogenous purines for ATP biosynthesis^123^. The need for nucleotide substrates increases substantially during inflammation, injury and wound healing, and consistently, disease severity correlated with loss of hypoxanthine in the epithelium in a chemically induced murine model of colitis^123^. In line, supplementation of hypoxanthine or selective colonization with purine-producing bacteria improved cellular energetics, promoted mucus generation and epithelial barrier function, resulting in enhanced wound healing and protection from colitis^122^. Under normal conditions, the intestinal microbiome produces and releases purines at high levels^123^, yet meeting the energy demand required for mucosal regeneration seems to be a bottle neck during active inflammation. Thus, supplementation of purines has been suggested as therapeutic approach to promote wound healing and remission in IBD patients^122,123^.

Metabolism of aromatic amino acids, particularly tryptophan, is another example for the tight interplay of microbiome and host metabolism^124^. Bacterial as well as host catabolism of the essential amino acid tryptophan gives rise to metabolites such as indoles and kynurenines, that are sensed by the aryl hydrocarbon receptor (Ahr)^87,125^. Ahr is a ligand-activated transcription factor located in the cytosol as well as inner mitochondrial membrane^126^ and impacts mitochondrial respiration and other cellular metabolic pathways^125,127^. Exerting various effects on epithelial and immune cells, Ahr-signaling orchestrates the key players of mucosal healing processes^128^. Activation of Ahr for example improves intestinal barrier function by modulating notch signaling^129^ and maintaining ISC homeostasis^130,131^. Concomitantly, Ahr activation induces regulatory T cells^132^, sustains IL-22 production in innate lymphoid cell (ILC) 3^133–135^ and enhances interleukin (IL) 10 receptor expression on epithelial cells^136^. These pathways are essential for intestinal wound healing and particularly important in the resolution of tissue damage^128^. Diets enriched in tryptophan^137^ or tryptophan-derived metabolites^131,136^ as well as enhanced bacterial tryptophan metabolism^135,138^ are shown to protect from inflammatory damage and accelerate wound healing. Conversely, microbiome-derived indole derivatives are diminished in IBD^104^, and this metabolic trait has been linked to reduced mucosal barrier integrity and disturbed immune balance under disease conditions^124^, suggesting these metabolites for prophylactic and therapeutic treatments^131^.

**Fig. 3:**
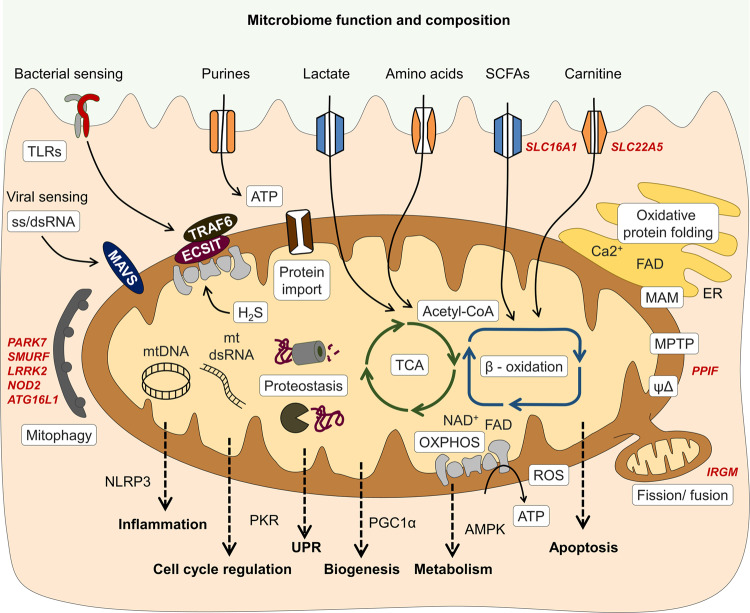
Intestinal epithelial cell mitochondria serve as metabolic signaling platform translating microbiome-derived signals into mucosal responses. Intestinal epithelial cells (IECs) sense the microbial environment via pattern recognition receptors including toll-like receptors (TLR) and take up diet and microbiota-derived metabolites. Activation of TLR signaling can impact the electron transport chain (ETC) and oxidative phosphorylation (OXPHOS) via TNF receptor associated factor (TRAF) 6 and ECSIT (Evolutionarily conserved signaling intermediate in Toll pathway), altering production of reactive oxygen species (ROS). Viral sensing involves mitochondrial antiviral-signaling protein (MAVS) and initiates inflammatory responses i.e., activating NFκB pathway. Microbiota-derived metabolites feed the tricarboxylic acid (TCA) cycle and mitochondrial beta oxidation or can enhance cellular energetics through salvage pathways (purines). Hydrogen sulfide (H2S) produced by bacteria can as electron donor for the ETC, but at high concentrations, inhibit Complex IV activity and other mitochondrial proteins. Mitochondria are embedded in an organelle network, in particular, an exchange of calcium, FAD, and ATP with the endoplasmic reticulum (ER) occurs at mitochondria-associated membranes (MAM) and is important for ER oxidative protein folding. Proteostasis, depending on protein import, chaperone activity, and proteases, is crucial to sustain mitochondrial functions, and disturbances of mitochondrial proteostasis are signaled by the mitochondrial unfolded protein response (UPRmt). Release of mitochondrial DNA and double-stranded (ds) mitochondrial RNA^184^ under stress conditions promotes inflammatory signaling and regulates cell cycle progression. Fission and fusion events as well as mitophagy are critical regulators of mitochondrial homeostasis and prevent accumulation of dysfunctional mitochondria and excess ROS production. Thus, mitochondria integrate environmental signals into metabolism, downstream employing various signaling pathways to contribute to cell fate decisions and determine cellular phenotypes. Gene names of known IBD risk variants involved in mitochondrial functions are given in dark red. AMPK AMP-activated protein kinase, NLRP3 NLR family pyrin domain containing 3, PGC1α Peroxisome proliferator-activated receptor gamma coactivator 1-alpha, PKR double-stranded RNA-activated protein kinase, SCFAs short chain fatty acids.

Reversely, microbiota-host interactions also contribute to disease susceptibility and progression. For instance, the bacterial metabolite hydrogen sulfide (H_2_S) is potentially harmful for IECs by inducing genotoxic damage and impairing mitochondrial OXPHOS^139,140^. In IBD, increased abundance of sulfate-reducing bacteria (i.e., H_2_S-producers) and in parallel, decreased expression of mitochondrial proteins involved in H_2_S- detoxification on host side have been reported^67^ and a recent integrated microbiota and metabolite profile-analysis linked Crohn’s disease activity to bacterial sulfur metabolism^12^ (Fig. 3). Thus, not only loss-of-function but also gain-of-function of the microbiome is crucial for IBD pathogenesis.

## Epithelial metabolic shaping of the microbiome

Several metabolic pathways are shared between microbiome and host, and common metabolites might originate from microbial or host metabolism. Hence, the question arises whether alterations in metabolite levels observed in the intestinal lumen under inflammatory conditions are due to microbiome alterations or mucosal dysfunction^141^. Tryptophan metabolism is a paradigm for shared and tightly entangled metabolic functions.

Intriguing aspects of host-microbiome interaction converging on tryptophan metabolism have been highlighted in Card9-deficient mice^104^, REG3A transgenic mice^142^ and Ido1-deficient mice^135^. Caspase recruitment domain family member 9 (*CARD9*) is a susceptibility gene for IBD and functions in the immune response against microorganisms. Host deficiency in Card9 or carrying *CARD9* risk alleles results in a microbiome with reduced capability to metabolize tryptophan into Ahr ligands, resulting in increased colitis susceptibility in the mouse model^104^. Vice versa, mice transgenic for the human secreted antimicrobial peptide REG3A, display altered composition of their microbiota, favoring L-Ornithine-producing lactobacilli. In turn, microbiome-derived L-Ornithine promotes generation of the Ahr ligand L-kynurenine in IECs, increasing ILC3 cell numbers and intestinal mucus formation^142^. Similarly, increased tryptophan availability in mice deficient in tryptophan-metabolizing indoleamine 2,3 dioxygenase-1 (Ido1) leads to an expansion of intestinal lactobacilli which use tryptophan instead of sugar as energy source. Lactobacilli produce the Ahr ligand indole-3-aldehyde and thereby contribute to mucosal IL-22 expression and colonization resistance to the fungus *Candida albicans*^135^. These findings exemplify the bi-directional cross-talk between host genetics/host-derived factors, and microbiome composition/function, and its relevance to disease.

Despite enormous efforts in cataloguing microbiome alterations^143^, the functional specificity and cause of dysbiosis is not well understood^144,145^. An intriguing new concept suggests that metabolic reprogramming of the intestinal epithelium contributes to the dysbiotic adaptation of microbial communities^146^, highlighting the bi-directional metabolic interaction of microbiome and host at the intestinal interface.

The underlying hypothesis is that epithelial metabolism is integral to the mechanisms used by the host to shape the microbiome for its own benefit^147^. To achieve this, differentiated colonocytes are supposed to preferentially oxidize butyrate and other fatty acids and employ OXPHOS, with oxidation of SCFAs accounting for approximately 70% of oxygen consumption in colonocytes^108^. As a result of high epithelial oxygen consumption, the epithelial surface is kept in a hypoxic state, favoring a microbiota dominated by obligate anaerobic bacteria. Thereby, oxygen depletion fosters bacteria converting fiber into fermentation products and making an otherwise non-usable energy source accessible to the host^146^. This mutualism is disrupted upon injury or inflammation, potentially causing a shift in colonocyte metabolism away from fatty acid oxidation, thereby reducing epithelial oxygen consumption. Parallel to the increase in oxygen emanating from the epithelial surface, luminal availability of by-products of the host inflammatory response such as nitrate, favors the growth of facultative anaerobes from the family of *Enterobacteriaceae*^146,148,149^. As outlined before, metabolic alterations cause proportional shifts in epithelial subtypes of the epithelium. Crypt hyperplasia, a common feature of IBD^150,151^, results from an expansion of transit amplifying cells and concomitantly involves a reduction of terminally differentiated epithelial cells, such as goblet cells^152^. These changes can be regarded as an excessive epithelial repair program. In comparison to terminally differentiated colonocytes, metabolism of transit amplifying cells is characterized by low oxygen consumption^153^, resembling alterations observed in host epithelial metabolism under inflammatory conditions.

Consistently, metabolic reprogramming of IECs offers new promising treatment options for IBD patients, targeting both, epithelial restitution and restoration of host-microbiome symbiosis.

## Epithelial – immune cell metabolic circuits

As mentioned above, tryptophan is catabolized by Ido1 in IECs as well as lamina propria immune cells, generating immunoregulatory kynurenine-based metabolites and resulting in tryptophan depletion. Under inflammatory conditions, such as IBD and GvHD but also in colorectal cancer (CRC), expression of Ido1 is strongly elevated^154,155^. Notably, the serum kynurenine to tryptophan ratio as a measure of Ido1 activity might be used as a disease biomarker^156^. The role of Ido1 and tryptophan metabolism, together with arginine metabolizing enzymes, has been extensively studied in the context of metabolic reprogramming of immune cells for balancing immunoregulatory and pro-inflammatory phenotypes^157–159^, thus being critical to wound healing, neoplasia, autoimmunity and the rejection of transplanted tissues^155,160–162^. Effects of Ido1 activity include promotion of T cell- mediated tolerance and antimicrobial effects^154^ and Ido1 might also act at the site of expression to decrease T-cell proliferation and survival, diminishing colonic inflammation and reducing disease severity^155^. Hence, the induction of Ido1 in intestinal epithelial cells under inflammatory conditions most likely represents an attempt to dampen immune cell activity and lower inflammation-associated tissue injury. In line, a subset of Paneth cells expressing Ido1 has recently been identified that might be involved in controlling immune responses towards the intestinal microbiome^163^. Comparing epithelial Ido1 expression with the extensive data available for the role of Ido1 in immune cells^161^ and considering the concept of competition for nutrients in the control of immune responses^164^, IECs potentially engage their metabolism to actively orchestrate mucosal immune responses. Determining the availability of metabolites in the lamina propria in conjunction with the microbiome, IECs might coordinate the recruitment of immune cells and guide immune cell functions to ensure efficient wound healing and barrier restitution upon mucosal injury.

Of note, immune cells and their cytokines reciprocally regulate IECs metabolism and function. For example, the pro-inflammatory cytokines TNF and IL-6, but also regulatory cytokines like IL-22 and IL-10 have been demonstrated to regulate mitochondrial metabolism^165–167^ as well as ISC activation and proliferation^168–170^. Moreover, Ido1 is induced by the pro-inflammatory cytokine interferon gamma, whereas anti-inflammatory cytokines including IL-10 and transforming growth factor (TGF) β inhibit Ido1, thus, activity of Ido1 in IECs reflects the balance between pro- and anti-inflammatory signals^171^. Collectively, this highlights IECs metabolism and mitochondria as interface of microbiome and immune cell-derived signals and underlines the potential of epithelial metabolism as therapeutic target.

Attempts to modify IEC metabolism, using P110, a small peptide inhibitor of mitochondrial fission^172^, or olaparib, a clinically applied PARP inhibitor improving mitochondrial function^173^, already succeeded in reducing chemically induced colitis in mice. Additionally, established drugs like 5-amino salicylic acid, that alters mitochondrial metabolism^174^, might already be efficient by targeting epithelial metabolism. Of note, metformin, first-line medication for the treatment of type 2 diabetes, strongly affects intestinal microbiome composition and function^175^, and in parallel, alters IEC metabolism^176^. With regard to the host-microbiome symbiosis outlined above, identification of specific microbiome-derived metabolites conferring to epithelial homeostasis could be a promising aim of future research.

## Open questions and conclusion

Metabolic fitness emerged as new frontier in intestinal epithelial homeostasis and disease pathogenesis, and multiple extrinsic as well as intrinsic factors converge at this junction. Mitochondrial dysfunction is an early event in IBD pathogenesis, preceding inflammatory tissue aberrations^8,18–21^. Yet, it still needs to be clarified of whether these alterations are cause or consequence in response to injury and inflammation. These findings and insights gained in the field of host-microbiome symbiosis added new dimensions to the old hypothesis of IBD being an “energy deficiency disease” of the intestinal epithelium, evolving the idea of epithelial metabolism as central gatekeeper of barrier integrity and mucosal tolerance.

We propose that intrinsic defects in cellular metabolism cause epithelial dysfunction (metabolic injury) evoking attempts of the ISC niche to reconstitute normal tissue architecture and function. Metabolic injury may cause aberrant tissue responses reminiscent of intestinal reconstitution and, failure to resolve metabolic injuries leads to incomplete tissue healing and persistence of focal inflammatory lesions, predisposing IBD patients to remitting disease phenotypes or even tumor formation. The underlying causes of metabolic injury are most likely highly individual, and comprise an interrelated portfolio of genetic susceptibility and triggers from the luminal environment, including diet and the microbiome^10,177–180^. Functional adaptations of the epithelium and in particular, the ISC niche are initiated by extrinsic signals affecting cellular metabolism. It remains largely elusive which factors targeting mitochondrial function control epithelial cell regeneration in response to metabolic disruption, and how these signals contribute to either healing and tissue homeostasis, or favor chronic inflammation or tumorigenesis.

More research is needed to elucidate the metabolic program of the intestinal interface, and its pathogenic role in the etiology of inflammatory and tumorigenic disorders. Next to metabolites involved in host-microbiome metabolic communication discussed in this review, there is a vast number of additional candidate pathways like vitamin B^181^ and lipid metabolism^182^ that converge on epithelial function^183^. Thus, a better molecular understanding of signals and mediators in regenerative tissue responses and resolution of metabolic injuries is critical to develop clinically-relevant therapeutic interventions focusing on the enforcement of epithelial regenerative capacity by improving metabolic fitness, a novel strategy for combating intestinal diseases.
